# Integration of Culture-Based and Molecular Analysis of a Complex Sponge-Associated Bacterial Community

**DOI:** 10.1371/journal.pone.0090517

**Published:** 2014-03-11

**Authors:** Naomi F. Montalvo, Jeanette Davis, Jan Vicente, Raquel Pittiglio, Jacques Ravel, Russell T. Hill

**Affiliations:** 1 Institute of Marine and Environmental Technology, University of Maryland Center for Environmental Science, Baltimore, Maryland, United States of America; 2 Institute for Genome Sciences, University of Maryland School of Medicine, Baltimore, Maryland, United States of America; California Department of Public Health, United States of America

## Abstract

The bacterial communities of sponges have been studied using molecular techniques as well as culture-based techniques, but the communities described by these two methods are remarkably distinct. Culture-based methods describe communities dominated by *Proteobacteria*, and *Actinomycetes* while molecular methods describe communities dominated by predominantly uncultivated groups such as the *Chloroflexi*, *Acidobacteria*, and *Acidimicrobidae*. In this study, we used a wide range of culture media to increase the diversity of cultivable bacteria from the closely related giant barrel sponges, *Xestospongia muta* collected from the Florida Keys, Atlantic Ocean and *Xestospongia testudinaria*, collected from Indonesia, Pacific Ocean. Over 400 pure cultures were isolated and identified from *X. muta* and *X. testudinaria* and over 90 bacterial species were represented. Over 16,000 pyrosequences were analyzed and assigned to 976 OTUs. We employed both cultured-based methods and pyrosequencing to look for patterns of overlap between the culturable and molecular communities. Only one OTU was found in both the molecular and culturable communities, revealing limitations inherent in both approaches.

## Introduction

Marine sponges (Phylum *Porifera*) and their microbial symbionts have garnered great interest in recent decades. Two reasons for this interest are 1) the study of sponge-microbe interactions as a model of complex symbioses and 2) pharmaceutical leads from natural products derived from sponges [1]. Some of these natural products may be produced, not by the sponge hosts, but by microbial symbionts of the sponges [2], [3]. While advances in sequencing technology have led to a deeper understanding of sponge microbial communities, the functional roles of these microbial populations has become the primary focus. Several studies have contributed to sponge symbiont function including nitrogen cycling [1], [4], [5], [6], carbon fixation, and vitamin B12 metabolism [7], [8]. All of these studies suggest bacteria living in association with sponges can provide specific nutrients. However, there are many unanswered questions surrounding the methods by which sponges acquire and maintain their symbionts, the effects of microbes on sponge health, and the specificity of the microbial symbionts to their sponge hosts. A complete understanding of the ecology and genetic diversity of these microbes and their roles in host health, and the production of natural products requires laboratory cultivation. It is now very apparent that many of the major groups of bacteria that are associated with sponges are not being grown in the laboratory. This finding is at once interesting and challenging.

Cultivation provides access to genetic and biochemical characteristics of each microorganism that may not be revealed by 16S rRNA gene metagenomics. Several bacterial strains were isolated from sponge *Halichondria* sp. collected from the Indo Pacific region with a wide range of antimicrobial activity revealing sponge isolates remain a rich source of novel secondary metabolites [9]. Esteves *et al.* detected for the first time polyketide synthase (PKS) genes in cultured bacteria from the genus *Aquimarina* (Bacteroidetes) isolated from Irciniidae sponges in the Northeast Atlantic [10]. Aside from the production of bioactive compounds, cultured sponge-associated isolates also reveal ecological relevance. The production of the quorum sensing signal molecule N-acyl homoserine lactones (AHLs) that are involved in cell-cell communication and bacterial colonization of higher organisms has been detected in the cultured community of sponge *Mycale laxissima* [11], [12]. A further study examined a specific symbiont of *M. laxissima*, *Ruegeria* sp. KLH11 and confirmed a complex quorum-sensing network activates flagellar motility and inhibits biofilm formation [13]. A culturable α-proteobacterial symbiont was obtained from several sponges from different locations [14], also suggesting the ecologically importance of this cultured bacterium.

Giant barrel sponges of the genus *Xestospongia* are prolific members of tropical reef environments. Bacterial 16S rRNA gene libraries from the sponges *Xestospongia muta* and *Xestospongia testudinaria* show that these closely related sponges are hosts to diverse bacterial communities that are maintained in both sponges from geographically distant locations and are dominated by *Chloroflexi*, *Acidobacteria* and *Actinobacteria* [15], [16]. In addition, 16S rRNA gene sequences from these sponges are more similar to previously published sponge-derived sequences than the 16S rRNA genes from the surrounding water [16]. These findings suggest a stable relationship between the sponges and their bacteria. Culturing efforts may provide additional insight into the bacteria associated with *Xestospongia* spp. In this study, we have employed a wide range of culture media to increase the diversity of culturable bacteria from *X. muta* and *X. testudinaria*. Also, pyrosequencing was used to obtain insights into these bacterial communities to determine whether many of the cultured isolates from these sponges are present at low numbers in the complex bacterial communities associated with these sponges. We found that only one OTU, classified as a *Gammaproteobacteria*, was present in the cultured and non-cultured bacterial community of *Xestospongia* spp.

## Materials and Methods

### Sponge collection

Sponges were collected generally as described by Montalvo and Hill [5]. Specifically, *X. muta* was collected at Conch Reef, Key Largo, Florida, USA (24°56.82′N, 80°27.40′W). Sponge samples were collected in June 2004 (Xm45, Xm49, and XmE), August 2005 (Xm51-Xm54, Xm56, and XmF), and October 2008 (Xm81-Xm83, Xm85 and Xm86). *X. testudinaria* was collected from Manado Bay, Indonesia (01°32′N, 124°55′E) in December 2005 (Xt01-Xt03, XtC and XtD). A 1-cm^3^ section of sponge tissue was reserved for bacterial cultivation and the remaining sponge tissue was immediately stored at −80°C for later DNA extraction for pyrosequencing. Permits and approval for the collections at Conch Reef, Florida were obtained from the Florida Fish and Wildlife Conservation Commission (Special Activity License Permit # 04SR-883 valid from 19 May 2004–18 May, 2007; Permit #08SR-833 valid from 26 August 2008–25 August 2011). Collections in Indonesia are part of a collaborative research project with Dr. Subagus Wahyuono at Gadjah Mada University in Indonesia and Dr. Mark Hamann at The University of Mississippi. Sponges were collected outside of protected areas and no local collection permits were required for these collections in 2005. Sponge samples were exported from Manado under the collaborative agreement between Gadjah Mada University and the University of Mississippi. These samples were imported into the US under US Fish and Wildlife Service declaration (USFWS Form 3-177 dated 12/15/2005). All necessary permits were obtained for the described study, which complied with all relevant regulations.

### Bacterial cultivation

Sponge samples were processed for bacterial cultivation within 2 hours of collection. A 1-cm^3^ section of sponge tissue was pulverized in 9 ml of sterile ASW. The homogenate was used to create a 10-fold dilution series of which 100 µl aliquots were added to solid or liquid media. Dilutions were plated on Marine Agar 2216 (MA2216) (Becton Dickinson) and incubated for 1–2 weeks for plate counts and dominant morphotypes. Initial plate counts and subcultivation of dominant morphotypes were done in field labs in Manado and Key Largo, 3–5 days after sample collection. Pure isolates of dominant morphotypes were transported back to the lab for further identification. Isolation plates were then resealed and incubated to allow for growth of additional slow growing bacteria. Dilutions were plated on the following additional media to increase the diversity of cultivable bacteria: 1/10 strength MA2216 with and without antimicrobials, Actinomycete Isolation Agar (Becton Dickenson) with antimicrobials, FSWFA medium (filter-sterilized seawater supplemented with a methanol extraction of lipids from lyophilized *X. muta* tissue, solidified with agarose), modified Glycerol-Asparagine Agar (peptone, 2 g/L; L-Asparagine, 0.5 g/L; Na propionate, 4 g/L; K_2_HPO_4_, 0.5 g/L; MgSO_4_×7H_2_O, 0.1 g/L; FeSO_4_, 0.001 g/L; Glycerol, 5 g/L; Agar, 18 g/L) with antimicrobials, ISP medium 2 (Becton Dickenson) with and without antimicrobials, NTM [17], [18] with vitamins, with and without antimicrobials, R2A medium [19] with antimicrobials, R2A with vitamins (R2AV) with and without antimicrobials, SN medium, Starch Casein Agar [20] with antimicrobials, VL55 medium [21] with and without antimicrobials, and whole seawater medium (WSWA) (filter-sterilized seawater with vitamins, solidified with agarose). All media without salt in their original formulations were supplemented with NaCl at 20 g/L. All media containing vitamins were supplemented with 1 ml/L Vitamin Solution 1 [22] and 3 ml/L Vitamin Solution 2 [22]. All media containing antimicrobials were supplemented with 10 µg/ml cycloheximide, 10 µg/ml nalidixic acid, and 25 µg/ml nystatin. These plates were incubated for six to nine weeks in dark conditions at 25°C and all distinct colony morphotypes were subcultured for further identification.

### Identification and classification of bacterial isolates

DNA was extracted from pure cultures using the Mo Bio UltraClean Microbial DNA Isolation Kit (Mo Bio Laboratories Inc, Carlsbad, CA) according to the manufacturer's instructions. 16S rRNA gene fragments were PCR-amplified with the universal primers 27F and 1492R and sequenced on an ABI 3130 XL Genetic Analyzer (Applied Biosystems, Foster City, CA). Sequences were assembled and edited using Pregap4 and Gap4 from the Staden Package (http://staden.sourceforge.net/). Sequences were classified with the RDP classifier. The nearest relatives for each sequence were obtained from the GenBank database using the blastn tool (http://blast.ncbi.nlm.nih.gov/) in May 2011.

### Pyrosequencing of barcoded 16S rRNA gene amplicons

Total DNA was extracted from lyophilized sponge tissue as described by Montalvo *et al.* [15]. Universal primers 27F and 338R were used for PCR amplification of the V1-V2 hypervariable regions of 16S rRNA genes as described by Ravel et al. [23]. Briefly, the 338R primer included a unique sequence tag to barcode each sample. The V1-V2 regions of 16S rRNA genes were amplified using AmpliTaq Gold DNA polymerase (Applied Biosystems) and 50 ng of template DNA in a total reaction volume of 50 µL. Reactions were run in a PTC-100 thermal controller (MJ Research). Negative controls without a template were included for each bar-coded primer pair. The presence of amplicons was confirmed by gel elec-trophoresis on a 2% agarose gel and staining with SYBRGreen. PCR products were quantified using a GelDoc quantification system (BioRad) and the Quant-iT PicoGreen dsDNA assay. Equimolar amounts (100 ng) of the PCR amplicons were mixed in a single tube. Amplification primers and reaction buffer were removed from each sample using the AMPure Kit (Agencourt). The purified amplicon mixtures were sequenced by 454 FLX pyrosequencing using 454 Life Sciences primer A by the Genomics Resource Center at the Institute for Genome Sciences, University of Maryland School of Medicine, using protocols recommended by the manufacturer as amended by the Center. Sequences were binned by sample as previously described [23]. The following criteria were used to assess the quality of the reads: sequence reads must include a perfect match to the unique sequence tag, be at least 200 bp in length, have no undetermined bases, and be at least a 60% match to a previously described 16S rRNA sequence.

### Estimation of microbial diversity and analysis of 454 sequences

Mothur (http://www.mothur.org/) [24] was used to assign sequences to operational taxonomic units (OTUs) and to generate rarefaction and rank abundance curves for observed OTUs. Sequences were clustered using the average neighbor algorithm at a distance of 0.03. The alignment and phylogenetic analysis was performed using ARB software package [25]. Sequences were classified using the SILVA rRNA database project [26].

### 16S rRNA gene sequence accession numbers

The bacterial 16S rRNA gene sequences obtained in this study have been deposited on the public MG-RAST server (http://metagenomics.anl.gov) under MG-RAST accession numbers 4551688.3 to 4551692.3. All sequences can be downloaded by accessing the *Xestospongia* spp. Associated Bacteria Study using the MG-RAST Project link (http://metagenomics.anl.gov/linkin.cgi?project=7756)

## Results and Discussion

### Diversity of cultured isolates

A total of 434 cultured bacterial isolates were identified from *X. muta* and *X. testudinaria*. From *X. testudinaria* samples, 50 isolates from three isolation media were identified from 5 sponges collected in 2005. From *X. muta* samples, 384 isolates from 18 isolation media were identified from 14 sponges collected in 2004, 2005, and 2008. A total of 50 genera were represented; 18 genera from the *Actinobacteria*, 7 genera from the *Firmicutes*, 12 genera of *Gammaproteobacteria*, 11 genera of *Alphaproteobacteria*, and 2 genera of *Bacteriodetes* (Table 1, Table S1 and Table S2).

**Table 1:** Cultured isolates from *X. muta* and *X. testudinaria*.

	*X. muta*		*X. testudinaria*			*X. muta*		*X. testudinaria*	
	Isolates	OTUs	Isolates	OTUs		Isolates	OTUs	Isolates	OTUs
**Gammaproteobacteria**	**116**	**23**	**3**	**3**	**Firmicutes**	**110**	**21**	**18**	**6**
*Endozoicomonas*	8	3	2	2	*Aneurinibacillus*	19	1	1	1
*Aestuariibacter*	1	1	0	0	*Brevibacillus*	6	1	0	0
*Agarivorans*	2	1	0	0	*Paenibacillus*	3	1	0	0
*Vibrio*	50	5	1	1	*Staphylococcus*	17	3	6	2
*Alteromonas*	13	1	0	0	*Bacillus*	61	13	9	2
*Ferrimonas*	1	1	0	0	*Lysinibacillus*	3	1	2	1
*Pseudoalteromonas*	23	6	0	0	*Virgibacillus*	1	1	0	0
*Shewanella*	1	1	0	0	**Actinobacteria**	**101**	**28**	**25**	**12**
*Photobacterium*	5	1	0	0	*Brevibacterium*	8	4	4	1
*Microbulbifer*	7	1	0	0	*Curtobacterium*	3	1	0	0
*Rheinheimera*	1	1	0	0	*Micromonospora*	26	3	4	3
*Pseudomonas*	4	1	0	0	*Rhodococcus*	5	1	2	2
**Alphaproteobacteria**	**54**	**14**	**4**	**3**	*Kocuria*	11	2	0	0
*Roseomonas*	1	1	0	0	*Micrococcus*	20	1	3	1
*Phenylobacterium*	1	1	0	0	*Rothia*	1	1	0	0
*Erythrobacter*	4	2	1	1	*Corynebacterium*	1	1	0	0
*Hoeflea*	2	1	0	0	*Brachybacterium*	2	1	0	0
*Labrenzia*	3	1	0	0	*Gordonia*	6	1	0	0
*Pseudovibrio*	35	1	3	2	*Streptomyces*	10	5	1	1
*Roseovarius*	1	1	0	0	*Dermacoccus*	2	1	0	0
*Silicibacter*	2	1	0	0	*Mycobacterium*	1	1	0	0
*Pelagibius*	1	1	0	0	*Arthrobacter*	2	1	5	2
*Sphingomonas*	3	3	0	0	*Leucobacter*	1	1	0	0
*Sphingopyxis*	1	1	0	0	*Nesterenkonia*	1	1	0	0
**Bacteroidetes**	**3**	**3**	**0**	**0**	*Nocardiopsis*	1	1	0	0
*Muricauda*	2	2	0	0	*Microbacterium*	0	0	6	2
*Winogradskyella*	1	1	0	0	**Total**	**384**	**88**	**50**	**24**

The bacterial phyla or classes are bolded and the genera are listed below each phylum or class. The number of isolates cultured and the number of OTUs from *X. muta* and *X. testudinaria* are listed for each bacterial group.

Seventeen genera of *Actinobacteria* were isolated from *X. muta*. To our knowledge, this is the most genera of *Actinobacteria* reported from a single sponge species, as well as the first report of *Nesterenkonia* sp. isolated from a marine sponge. *Actinobacteria* were isolated on 15 of the 18 media used in this study, with the most diversity coming from R2A supplemented with vitamins and antimicrobials (Table S1). The cultured *Actinobacteria* were dominated by *Micromonospora* sp., which are commonly found in sponges and have been shown to produce natural products [27], [28]. *Micrococcus*, *Brevibacterium*, *Kocuria*, and *Streptomyces* followed in abundance in the assemblage of cultured actinomycetes, all of which are commonly found in sponges. *Microbacterium* spp., which have been found previously in sponges [29], were the dominant *Actinobacteria* cultured from *X. testudinaria*, but they were not isolated from *X. muta*.

The *Gammaproteobacteria* were dominated by *Vibrio* spp. and *Pseudoalteromonas* spp. The *Firmicutes* were dominated by *Bacillus* spp. The cultured *Alphaproteobacteria* were dominated by isolates by a single *Pseudovibrio* sp. that are 99% identical to the NW001-like *Alphaproteobacteria* first discovered by Webster and Hill [20], and shown to dominate the culturable community of the marine sponge *Rhopaloeides odorabile*. These bacteria were further characterized by Enticknap *et al.* [14] and were found to be present in sponges from several oceans, as well as in the larvae of *Mycale laxissima*. The presence of these NW001-like *Alphaproteobacteria* in both *X. muta* and *X. testudinaria* sponges, further strengthens indications that these bacteria are sponge-specific symbionts.

All *Bacteroidetes* belong to the class *Flavobacteria*. Two novel *Flavobacteria* were isolated from *X. muta*. Isolates SN8107F and SN8106F were 95% identical to their closest relatives *Coccinimonas marina* and *Muricauda* sp. BB-My12.

### Diversity of 454 sequences

**Figure 1:**
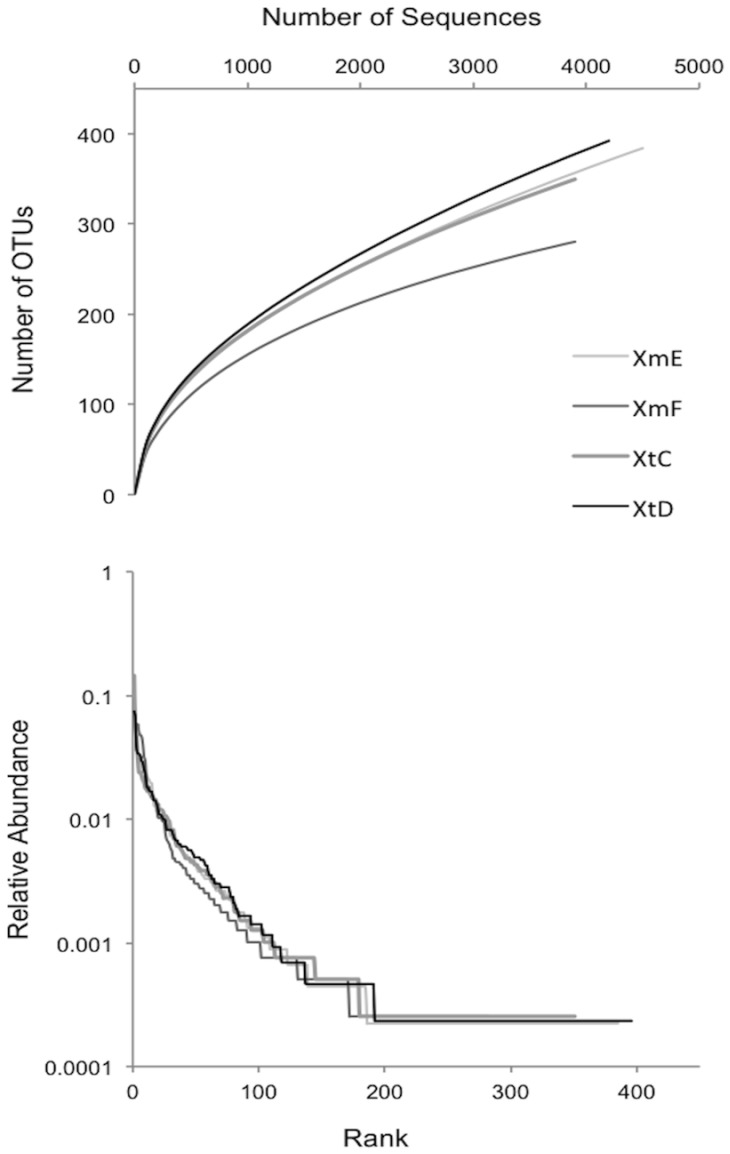
Diversity of *Xestospongia*-associated bacterial 16S rRNA gene fragments. Rarefaction curves (top) and rank-abundance curves (bottom) showing the diversity of sequences from two individuals of *X. testudinaria* (XtC and XtD) and *X. muta* (XmE and XmF).

**Figure 2:**
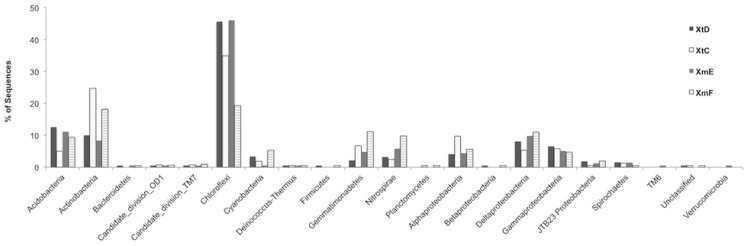
Taxonomic distribution of *Xestospongia*-associated bacterial 16S rRNA gene fragments at 3% OTUs. Shown are percent sequences for two individuals of *X. testudinaria* (XtC and XtD) and two individuals of *X. muta* (XmE and XmF). Values >0.4% are shown as 0.4% for clarity.

**Figure 3:**
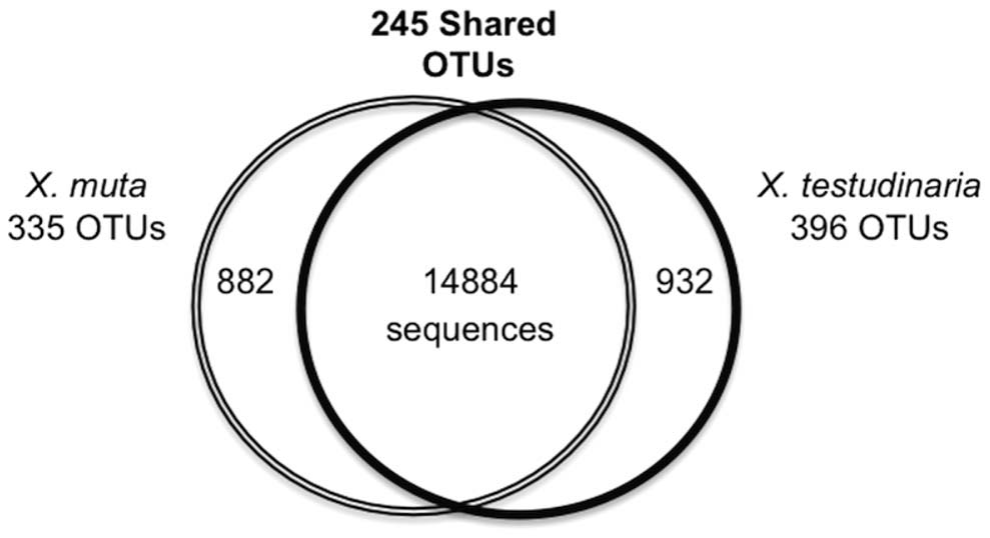
Culture-independent analysis of *Xestospongia*-associated 16S rRNA gene fragments. Shown are the numbers of OTUs shared between the cultured-independent community of *X. muta* and *X. testudinaria*. The numbers of sequences from each sample are represented inside the circles.

A total of 16,689 16S rRNA gene sequences spanning the V1-V2 variable regions were analyzed from four different sponge individuals, two *X. muta* (XmE and XmF) and two *X. testudinaria* (XtC and XtD). Of the 16,689 sequences, 5,406 were unique and were assigned to 976 OTUs (Fig. 1). These sequences were classified as *Acidobacteria, Actinobacteria, Bacteroidetes*, Candidate division OD1, Candidate division TM7, *Chloroflexi, Cyanobacteria, Deinococcus-Thermus, Firmicutes, Gemmatimonadetes, Nitrospirae, Planctomycetes, Alphaproteobacteria, Betaproteobacteria, Deltaproteobacteria, Gammaproteobacteria*, JTB23 Proteobacteria, *Spirochaetes*, *TM6, Verrucomicrobia* and unclassified bacteria (Fig. 2). *Chloroflexi*, *Actinobacteria* and *Acidobacteria* were the dominant bacterial groups. Over 85% of the sequences fell into 245 OTUs shared between *X. muta* and *X. testudinaria* (Fig. 3). Of the shared OTUs between samples, 70 OTUs contained sequences from all four sponge individuals and the majority of these OTUs were related to sequences that are sponge-associated, (47 OTUs) or sponge-coral associated (21 OTUs) (Table S3).

Only six of the 20 classified bacterial groups represented in the 454 sequence data, did not include OTUs shared by all four sponges (*Bacteroidetes, Firmicutes, Planctomycetes, Betaproteobacteria, TM6*, and *Verrucomicrobia*) and these groups were only present in very low numbers, with each group representing less than 1% of the total sequence data (Fig. 2).

### Overlap of 454 sequences and cultured isolates

**Figure 4:**
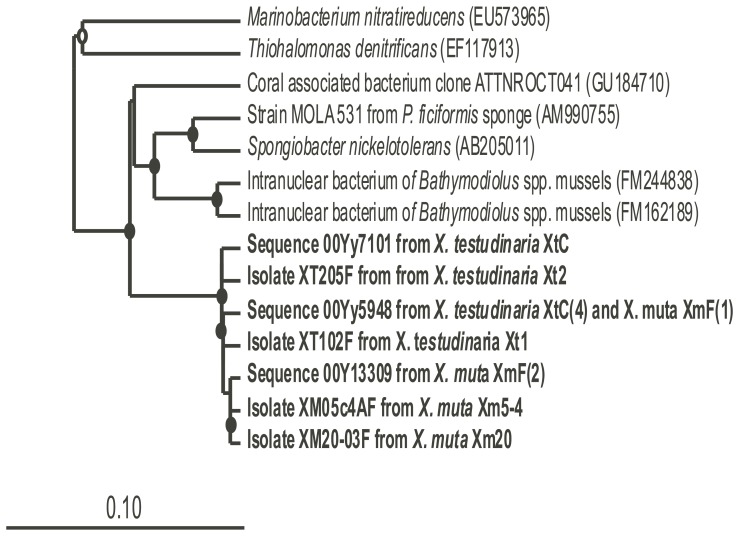
Phylogenetic tree of shared OTUs in the culture-based and culture-independent analysis of *Xestospongia* spp. Sequences from this study are bolded and their closest relatives are included. Bootstrap values (neighboring-joining method, 100 replicates) are indicated by closed circles (values >90%) and open circles (values >75%). The scale bar represents 10% sequence divergence.

*Gammaproteobacteria* were the only group of cultured isolates also represented in the 454 sequences. Four *Gammaproteobacteria* of the class *Oceanospirillales* were isolated from two *X. muta* sponges and two *X. testudinaria* sponges on MA2216 and also seen in the 454 sequences of XtC and XmF. These isolates were 91–93% identical to their closest relatives in GenBank, *Spongiobacter nickelotolerans* and strain MOLA 531, and 97–99% identical to each other. These sequences formed a single OTU and may represent a novel group of culturable sponge symbionts (Fig. 4). *Spongiobacter* sp. have been found in association with sponges and corals [30], [31], [32]. One study examined coral-associated bacteria in their role in biogeochemical cycling of sulfur and found that the genus *Spongiobacter* sp. was one of the four genera isolated on media with either dimethlysulfoniopropionate (DMSP) or dimethyl sulfide (DMS) as the sole carbon source from two coral species suggesting that these bacteria have the potential to metabolize sulfur compounds and may contribute to coral health [33]. Consistent with other studies, this cultured strain could potentially provide nutrients to the sponges. Although there was only one shared OTU between the cultured and pyrosequenced community, *Spongiobacter* sp. is a well documented isolate of sponges [1] and strain MOLA is also isolated from a sponge, which again supports sponge-specific symbionts. Further study of these bacteria is warranted, as well as looking for these organisms in other marine sponges.

## Conclusion

It is not surprising that the deep pyrosequencing analysis of the sponges *X. muta* and *X. testudinaria* revealed many major groups of bacteria that do not yet have any cultured representatives. Bacterial symbionts can have ancient relationships with their hosts and live within specialized structures or highly enriched host tissue, making them difficult to culture [34]. Sponges represent the oldest metazoan phylum [35] and the continuance of bacteria associated with sponges can suggest a long and stable relationship. Several studies have highlighted the large taxonomic differences between the culture dependent and culture independent communities [34], [36], [37], [38]. However, several groups have increased culturing efforts of microbes associated with sponges. Wilkinson *et al.* pioneered this effort and found a similar sponge-specific bacterial isolate that was found in 9 out of 10 sponges samples from both the Mediterranean and Great Barrier Reef [39]. Several studies have noted cultured sponge-specific isolates from many marine sponges in difference locations [40], [41]. A recent survey of the largest cultured collection of bacteria associated with marine sponges uncovered sponge specific isolates that were previously reported as “uncultured” however; nearly 88% of the isolates were dominated by the commonly cultivable class *Gammaproteobacteria* [42]. *Gammaproteobacteria* were also one of the dominant cultured members in the present study.

Our previous work in molecular analysis of bacteria associated with *Xestospongia* sponges [15], [16] already partially revealed the remarkable bacterial diversity associated with these sponges, specifically the abundance of *Chloroflexi* and *Actinobacteria*. The pyrosequencing reported here was consistent with our previous findings with respect to the abundance bacterial groups and extended this work with an additional 27 OTUs that were present in all four sponge individuals, but were not previously detected in the 16S rRNA clone library analysis [16], including sequences classified as candidate division OD1 (Table S3). The presence of OTUs found in both *Xestospongia* individuals and different species from geographically distant location, may not just suggest a possible sponge-specific community but a sponge-species-specific community. *Lee* et al. observed several Red Sea sponges possessed a highly sponge-specific or even sponge-species-specific microbial community that are resistant to environmental disturbance [43]. Another study used pyrosequencing on 32 sponges in 8 different locations and found a minimal core bacterial community that existed in all sponges but a large species-specific bacterial community associated with the sponges [44]. The bacterial community associated with *A. corrugata* was assessed between two seasons and although there were slight shifts in the community the overall species-specific bacteria remained [45]. The current study contributes to the increasing knowledge of sponge bacterial symbionts and the growing notion of a sponge-species-specific bacterial community. There is still much work needed to understand the roles of these bacterial symbionts and recent work using metaproteogenomics provided new insights into the interactions of sponge-associated microbial communities [46].

It is more surprising that the extensive culture-based work undertaken here resulted in the isolation of many groups of bacteria that are not detected by even the deep pyrosequencing community analysis. There are two possible explanations for this. First, the cultured bacteria may be present as only very minor constituents of the sponge-associated bacterial community. Many of these bacterial groups were isolated repeatedly from different individuals of the same sponge species, and often from both *Xestospongia* spp. sponges. This suggests that they are consistently associated with the sponges and may play an important role in the bacterial community, even if they are present at very low numbers. A recent study examined the cultured and uncultured bacteria associated with soil samples and observed soil bacteria captured by culturing were in low abundance or absent in the culture-dependent community, revealing a fraction of a “rare biosphere” that would remain undiscovered by pyrosequencing at a given sampling depth [47]. The second possibility is that the 16S rRNA community analysis [16] and the pyrosequencing analysis may have significant biases resulting in the lack of detection of entire bacterial groups. This concern has been previously discussed [48], [49], [50], [51]. Approaches to investigate this possibility include FISH targeting of bacteria found in culture based work (e.g. the *Vibrio*, *Pseudovibrio*, *Micrococcus*, and *Micromonospora* spp., that were often cultured from *X. muta* and *X. testudinaria*), and PCR detection with specific primers designed to target these groups, followed by RT-PCR or FISH to assess their numbers. Cassler *et al.* used RT-PCR to quantify the cultured and uncultured bacteria associated with a marine sponge and found three uncultured members in large quantities and only detected one of three cultured members at a trace level [52]. The present study highlights cultured members isolated from different individuals of the same sponge species as well as different species within the genus *Xestospongia*. Therefore using RT-PCR for cultured groups associated with *Xestospongia* spp. that have shown a consistent associated may reveal higher quantities of the bacteria.

This work and other recent studies [53], [54] emphasize the importance of extensive culture-based studies for isolation of representatives of the novel bacterial groups found in marine sponges. In this study a very diverse group of bacteria was cultured from *Xestospongia* sponges, but very little overlap was found between the culturable community and the community revealed by molecular approaches. Several novel isolates were cultured, however, the majority of the bacteria that were cultured are closely related to organisms that have been found in other environments, while the majority of the sequences in the molecular analysis have been found only in sponges, or sponges and corals. As previously noted, culturing is adding new insight about sponge bacteria symbionts. However, there is still much effort needed to culture representatives of many of the key groups of bacteria found in marine sponges and one of the challenges for the field of sponge microbiology, as highlighted by Taylor *et al.* [54], is to successfully culture representatives of these groups.

## Supporting Information

Table S1 Cultured isolates from *X. muta*. Cultured isolates from *X. muta*, their closest relatives based on 16S rRNA gene sequence analysis (GenBank) and the isolation medium types are listed.
(PDF)

Table S2 Cultured isolates from *X. testudinaria*. Shown are cultured isolates from *Xestospongia testudinaria*, their closest relatives based on 16S rRNA gene sequence analysis (GenBank) and the isolation medium types.
(PDF)

Table S3 OTUs shared by all individuals of *X. muta* and *X. testudinaria*, based on 16S rRNA gene sequence analysis. 70 OTUs were shared by all individuals of the two *Xestospongia* sp. Triangles indicate OTUs that were *Xestospongia*-specific. Open squares indicate OTUs that were sponge-specific. Circles indicate OTUs that were sponge/coral specific. Closed squares indicate OTUs that were not specific. Asterisks indicate OTUs that were found in a previous study of *X. muta* and *X. testudinaria* [16].
(PDF)
