# Supplementary material for: Integration of Culture-Based and Molecular Analysis of a Complex Sponge-Associated Bacterial Community
Source: PLoS One. 2014 Mar 11;9(3):e90517. doi: 10.1371/journal.pone.0090517 (PMC3949686; doi:10.1371/journal.pone.0090517)
Supplement: Table S1 — Cultured isolates from X. muta. Cultured isolates from X. muta, their closest relatives based on 16S rRNA gene sequence analysis (GenBank) and the isolation medium types are listed. (PDF) [file pone.0090517.s001.pdf]

Isolate	Closest Relative	Acc. No.	% Identity	SpongeID	Isolation Medium
		Actinomycetes			
XM4189C2	*Arthrobacter luteolus*	DQ486130	99	Xm54	R2AV+abx
XM5069A	*Arthrobacter luteolus*	DQ486130	99	XmF	R2AV+abx
XM85132B	*Brachybacterium* sp. CJ-6	HQ455045	99	Xm85	FSWFA
XM4261	*Brachybacterium* sp. RS110	EU912470	100	Xm54	NTM+abx
M108201	*Brevibacterium* sp. SC9	EU099382	99	Xm82	1/10MA
XM4083	*Brevibacterium* sp. N78(2010)	HQ188605	99	Xm54	1/10MA+abx
XM1008	*Brevibacterium casei*	GQ284451	99	Xm51	ISP2+abx
XM5004	*Brevibacterium* sp. MJ24	GQ250446	99	XmF	ISP2+abx
XM6004	*Brevibacterium* sp. MN-6-a	AB609748	98	Xm56	ISP2+abx
R8603A2-F	*Brevibacterium* sp. M1-12	AM981204	99	XM86	R2AV
R8603B1-F	*Brevibacterium* sp. M1-12	AM981204	99	XM86	R2AV
R8603B2	*Brevibacterium* sp. M1-12	AM981204	99	XM86	R2AV
R8614	*Corynebacterium accolens*	AJ439346	99	XM86	R2AV+abx
M108304	*Curtobacterium citreum*	FJ544324	100	XM83	1/10MA
M108302	*Curtobacterium* sp. AeL09	EU741014	99	XM83	1/10MA
R8306BF	*Curtobacterium* sp. Fek20	EU741030	99	XM83	R2AV
XM1011-F	*Dermacoccus* sp. Ellin185	AF409027	99	Xm51	ISP2+abx
XM1017	*Dermacoccus* sp. Ellin185	AF409027	99	Xm51	ISP2+abx
XM4007-1	*Gordonia lacunae*	GU727686	99	Xm54	ISP2+abx
XM4153	*Gordonia* sp. HPCPW46	HM072350	98	Xm54	ISP2+abx
XM04c93	*Gordonia terrae*	FJ536292	99	Xm49	ISP2+abx
XM4006	*Gordonia terrae*	EU333873	100	Xm54	ISP2+abx
XM5112B	*Gordonia terrae*	EU333873	99	XmF	R2AV+abx
XM5122	*Gordonia* sp. G1	FJ939311	100	XmF	StarchCasein
XM6007	*Kocuria palustris*	HQ256825	99	Xm56	ISP2+abx
XM6008	*Kocuria palustris*	FR691399	99	Xm56	ISP2+abx
XM6009	*Kocuria palustris*	FR691399	99	Xm56	ISP2+abx
XM6011	*Kocuria palustris*	FR691399	100	Xm56	ISP2+abx
XM5008	*Kocuria rhizophila*	AY030315	99	XmF	ISP2+abx
R8201B-F	*Kocuria palustris*	EU333884	99	Xm82	R2AV
R8202A2-F	*Kocuria palustris*	EU333884	99	Xm82	R2AV
R8202A3-F	*Kocuria palustris*	EU333884	99	Xm82	R2AV
R8203B	*Kocuria palustris*	HM355687	99	Xm82	R2AV
XM5075B	*Kocuria* sp. 104	GQ352404	98	XmF	R2AV+abx
XM5075A	*Kocuria* sp. MH134	FJ626626	99	XmF	R2AV+abx
XM5003	*Leucobacter tardus*	HQ154561	98	XmF	ISP2+abx
XM-21-10	*Micrococcus luteus*	HM640421	98	Xm49	AIA
XM4016	*Micrococcus luteus*	AB617561	100	Xm54	ISP2+abx
XM1001	*Micrococcus* sp. MG-2010-D12	FR750272	100	Xm51	ISP2+abx
XM5007	*Micrococcus* sp. MG-2010-D12	FR750272	99	XmF	ISP2+abx
XM6010	*Micrococcus* sp. WB18-01	GU595336	100	Xm56	ISP2+abx
XM-20-06-F	*Micrococcus* sp. Y14C	EF175876	99	Xm49	ISP2+abx
XM6013-F	*Micrococcus yunnanensis*	FJ214355	99	Xm56	ISP2+abx
XM85A	*Micrococcus luteus*	AB617561	99	Xm85	MA2216
XM83A	*Micrococcus* sp. Bg-6	HQ916746	100	XM81	MA2216
XM81B	*Micrococcus* sp. MG-2010-D12	FR750272	100	XM81	MA2216
XM86B	*Micrococcus* sp. WB20-02	GU595337	100	XM86	MA2216
R8602A	*Micrococcus luteus*	EU071591	99	XM86	R2AV
R8601A	*Micrococcus* sp. BBN3T-03d	FJ357613	99	XM86	R2AV
R8601B	*Micrococcus* sp. MG-2010-D12	FR750272	100	XM86	R2AV
R8202B1-F	*Micrococcus* sp. PA-E028	FJ233852	100	Xm82	R2AV
R8202B2-F	*Micrococcus* sp. PA-E028	FJ233852	99	Xm82	R2AV
R8502A	*Micrococcus* sp. WB20-02	GU595337	100	Xm85	R2AV
R8602BF	*Micrococcus* sp. WT108	GQ152137	99	XM86	R2AV
XM4230A	*Micrococcus* sp. WB20-02	GU595337	99	Xm54	VL55+abx
XM4230B	*Micrococcus* sp. WB20-02	GU595337	99	Xm54	VL55+abx
XM-07-01	*Micromonospora* sp. M42	EF513641	97	XmE	MA2216
XM-20-01	*Micromonospora* sp. M42	EF513641	97	Xm49	MA2216
XM-21-01	*Micromonospora* sp. M42	EF513641	99	Xm49	MA2216

XM04c77	*Micromonospora* sp. 16	FJ205721	99	XmE	R2A
XM04c92	*Micromonospora* sp. 16	FJ205721	99	Xm49	R2A
XM04c205	*Micromonospora* sp. M42	EF513641	99	Xm49	R2A
R8504-F	*Micromonospora* sp. CNS-682_SD06	EU214969	99	Xm85	R2AV
R42004	*Micromonospora* sp. 10511	FJ216457	97	Xm49	R2AV+abx
R42010	*Micromonospora* sp. 10511	FJ216457	100	Xm49	R2AV+abx
R42101B	*Micromonospora* sp. 10511	FJ216457	99	Xm49	R2AV+abx
R45602	*Micromonospora* sp. 10511	FJ216457	99	Xm45	R2AV+abx
R42002	*Micromonospora* sp. 206203	EU437824	99	Xm49	R2AV+abx
R42003	*Micromonospora* sp. 206203	EU437824	99	Xm49	R2AV+abx
R42012	*Micromonospora* sp. 206203	EU437824	100	Xm49	R2AV+abx
R42013	*Micromonospora* sp. 206203	EU437824	99	Xm49	R2AV+abx
R42106	*Micromonospora* sp. 206203	EU437824	100	Xm49	R2AV+abx
R45604	*Micromonospora* sp. 206203	EU437824	99	Xm45	R2AV+abx
R45613	*Micromonospora* sp. 206801	EU437830	99	Xm45	R2AV+abx
R45606	*Micromonospora* sp. FXJ6.144	GU002092	99	Xm45	R2AV+abx
R45612	*Micromonospora* sp. FXJ6.144	GU002092	98	Xm45	R2AV+abx
XM3080A	*Micromonospora* sp. FXJ6.350	JF346470	98	Xm53	R2AV+abx
R42014A	*Micromonospora* sp. M42	EF513641	99	Xm49	R2AV+abx
R42102	*Micromonospora* sp. M42	EF513641	99	Xm49	R2AV+abx
R42105	*Micromonospora* sp. M42	EF513641	100	Xm49	R2AV+abx
R45601	*Micromonospora* sp. M42	EF513641	99	Xm45	R2AV+abx
R45610	*Micromonospora* sp. NN271	GU723672	99	Xm45	R2AV+abx
XM3072	*Mycobacterium* sp. Site1-3A	JF304592	99	Xm53	R2AV+abx
XM5085	*Nesterenkonia lacusekhoensis*	HQ202846	99	XmF	VL55+abx
XM6039	*Nocardiopsis umidischolae*	EU849610	99	Xm56	1/10MA+abx
R8101-F	*Rhodococcus* sp. SCSIO 00026	GQ871747	100	XM81	R2AV
R8102B-F	*Rhodococcus* sp. SCSIO 00026	GQ871747	99	XM81	R2AV
R8302A-F	*Rhodococcus* sp. SCSIO 00026	GQ871747	99	XM83	R2AV
R8302BF	*Rhodococcus* sp. SCSIO 00026	GQ871747	99	XM83	R2AV
R8303-F	*Rhodococcus* sp. SCSIO 00026	GQ871747	100	XM83	R2AV
R8613	*Rothia aeria*	EU293888	99	XM86	R2AV
XM6014	*Streptomyces* sp. HBUM 79010	EU119189	99	Xm56	1/10MA+abx
XM4025	*Streptomyces* sp. DRL40	FJ853198	99	Xm54	ISP2
XM4011	*Streptomyces* sp. FXJ6.329	JF346460	99	Xm54	ISP2+abx
XM3011	*Streptomyces* sp. HP11	GQ867031	100	Xm53	ISP2+abx
XM4009-F	*Streptomyces* sp. SFKS-14	HQ386729	99	Xm54	ISP2+abx
XM5125	*Streptomyces* sp. 11025	FJ262960	99	XmF	MA2216
XM05c5B	*Streptomyces tendae*	EU741192	99	XmF	MA2216
XM83C	*Streptomyces thermocoprophilus*	AB249938	99	XM81	MA2216
XM3086	*Streptomyces* sp. HBUM 79010	EU119189	99	Xm53	VL55
XM4193	*Streptomyces* sp. TFS 73	EF209051	99	Xm54	VL55
	Alphaproteobacteria				
XM6032	*Bacterium da*SW.34	EU935309	99	Xm56	1/10MA+abx
SN8108	*Erythrobacter longus*	AM691106	99	XM81	SN
SN8109	*Erythrobacter longus*	AM691106	100	XM81	SN
SN8111	*Erythrobacter longus*	AM691106	99	XM81	SN
XM860F	*Mesorhizobium* sp. GC15	AY690680	96	XM86	MA2216
R8308-F	*Mesorhizobium* sp. GC15	AY690680	97	XM83	R2AV
XM3105	*Marine bacterium 'Isolate 5'*	AY082665	98	Xm53	NTM
SN8103	Mucus bacterium 71	AY654812	99	XM81	SN
SN8113-F	*Stappia* sp. M4	AY307928	98	XM81	SN
SN8112B-F	Bacterium DG1026	AY258098	98	XM81	SN
XM6035	*Phenylobacterium falsum*	AJ717391	98	Xm56	1/10MA+abx
XM4244	*Alphaproteobacterium JE041*	DQ097262	99	Xm54	1/10MA
XM4242A	*Alphaproteobacterium JE066*	DQ097263	99	Xm54	1/10MA
XM4242B	*Alphaproteobacterium JE066*	DQ097263	99	Xm54	1/10MA
XM4246	*Alphaproteobacterium JE066*	DQ097263	100	Xm54	1/10MA
XM4063	Bacterium 1H203	JF411464	99	Xm54	1/10MA
XM4065	Bacterium 1H203	JF411464	99	Xm54	1/10MA
XM4069	Bacterium 1H203	JF411464	99	Xm54	1/10MA

XM4071	Bacterium 1H203	JF411464	99	Xm54	1/10MA
XM4064	Bacterium 1H215	JF411476	100	Xm54	1/10MA
XM4243	Bacterium 1H215	JF411476	99	Xm54	1/10MA
XM4247C	Bacterium 2D803	JF411488	100	Xm54	1/10MA
XM4248-F	Bacterium 2D803	JF411488	100	Xm54	1/10MA
FF8670	*Alphaproteobacterium F04*	DQ227656	99	XM86	FSW+FA
FW8621A	*Alphaproteobacterium JE041*	DQ097262	99	XM86	FSW+FA
FF8101	Bacterium 2D803	JF411488	100	XM81	FSW+FA
FF8107	Bacterium 2D803	JF411488	100	XM81	FSW+FA
FF8112	Bacterium 2D803	JF411488	100	XM81	FSW+FA
FF8113	Bacterium 2D803	JF411488	100	XM81	FSW+FA
FF8117	Bacterium 2D803	JF411488	100	XM81	FSW+FA
FF8666	Bacterium 2D803	JF411488	100	XM86	FSW+FA
FW8625	Bacterium 2D803	JF411488	99	XM86	FSW+FA
XM81107Z	*Alphaproteobacterium F04*	DQ227656	99	XM81	FSWFA
XM81114B	Bacterium 2D803	JF411488	99	XM81	FSWFA
XM05c4B	*Alphaproteobacterium JE065*	DQ097241	99	Xm54	MA2216
XM05c4D	Bacterium 2D803	JF411488	99	Xm54	MA2216
R8305	Bacterium 1H216	JF411477	99	XM83	R2AV
SN8102	Bacterium 2D803	JF411488	100	XM81	SN
XM4225A	*Alphaproteobacterium JE066*	DQ097263	99	Xm54	VL55+abx
W5502A	*Alphaproteobacterium F04*	DQ227656	99	XmF	WSWA
W5506	*Alphaproteobacterium F20*	DQ227657	99	XmF	WSWA
W5503	Bacterium 1H206	JF411467	99	XmF	WSWA
W5501F	Bacterium 1H215	JF411476	100	XmF	WSWA
W5502B	Bacterium 1H215	JF411476	100	XmF	WSWA
W5504	Bacterium 1H215	JF411476	99	XmF	WSWA
W5502C	Bacterium 2D803	JF411488	100	XmF	WSWA
XM5115	*Roseomonas cervicalis*	AF533353	99	XmF	1/10MA+abx
XM05c6A2	*Alphaproteobacterium C49*	AB330821	98	Xm56	MA2216
FF8669	*Rhodobacteraceae bacterium N04ML2*	EF629848	100	XM86	FSW+FA
XM05c6C	Ruegeria sp. 7PC-10	EF657806	98	Xm56	MA2216
M108303	*Sphingomonas yunnanensis*	EU730917	99	XM83	1/10MA
XM81120B	*Sphingomonas* sp. PA225	AM900788	100	XM81	FSWFA
XM3002	*Sphingomonadaceae bacterium NR203*	DQ520831	100	Xm53	ISP2+abx
XM4291	*Sphingopyxis* sp. SM105	EF424407	99	Xm54	NTM
	Bacilli				
XM6022-F	*Aneurinibacillus migulanus*	AB112723	99	Xm56	1/10MA+abx
XM6026	*Aneurinibacillus migulanus*	AB112723	99	Xm56	1/10MA+abx
XM3009	*Aneurinibacillus migulanus*	AB112723	99	Xm53	ISP2+abx
XM4012	*Aneurinibacillus migulanus*	AB112723	99	Xm54	ISP2+abx
XM5014	*Aneurinibacillus migulanus*	AB112723	99	XmF	ISP2+abx
XM3010	Bacterium C-TJ29	EU637636	98	Xm53	ISP2+abx
XM3114	*Aneurinibacillus migulanus*	AB112723	99	Xm53	NTM+abx
XM04c91	*Aneurinibacillus migulanus*	AB112723	99	Xm49	R2A
XM04c96	*Aneurinibacillus migulanus*	AB112723	99	Xm49	R2A
R42101A	*Aneurinibacillus migulanus*	AB112723	99	Xm49	R2AV+abx
XM3072F	*Aneurinibacillus migulanus*	AB112723	99	Xm53	R2AV+abx
XM3074B	*Aneurinibacillus migulanus*	AB112723	99	Xm53	R2AV+abx
XM3076A	*Aneurinibacillus migulanus*	AB112723	99	Xm53	R2AV+abx
XM4184B	*Aneurinibacillus migulanus*	AB112723	99	Xm54	R2AV+abx
XM4185A	*Aneurinibacillus migulanus*	AB112723	99	Xm54	R2AV+abx
XM4185D	*Aneurinibacillus migulanus*	AB112723	99	Xm54	R2AV+abx
XM4189B	*Aneurinibacillus migulanus*	AB112723	99	Xm54	R2AV+abx
XM4195A	*Aneurinibacillus migulanus*	AB112723	99	Xm54	VL55
XM4223	*Aneurinibacillus migulanus*	AB112723	99	Xm54	VL55+abx
XM4059-2	*Bacillus aryabhattai*	HQ242772	99	Xm54	1/10MA
M108302A	*Bacillus* sp. 2BSG-MG-22	AB533800	99	XM83	1/10MA
M108302B	*Bacillus* sp. 2BSG-MG-22	AB533800	99	XM83	1/10MA
M108304A	*Bacillus* sp. 2BSG-MG-22	AB533800	99	XM83	1/10MA
XM1022	*Bacillus licheniformis*	EU847237	99	Xm51	1/10MA+abx

XM4087	*Bacillus licheniformis*	HM753634	99	Xm54	1/10MA+abx
XM6018	*Bacillus licheniformis*	HQ917117	100	Xm56	1/10MA+abx
XM6020	*Bacillus licheniformis*	HQ143565	100	Xm56	1/10MA+abx
XM6021	*Bacillus licheniformis*	FJ493053	99	Xm56	1/10MA+abx
XM1023	*Bacillus* sp. CNJ826 PL04	DQ448748	99	Xm51	1/10MA+abx
XM4085	*Bacillus* sp. DB49(2010)	HM566930	99	Xm54	1/10MA+abx
XM6017	*Bacillus* sp. NQ18	EU919210	99	Xm56	1/10MA+abx
XM6016-2	*Bacillus* sp. SC83(2010)	HM566615	99	Xm56	1/10MA+abx
XM4082	*Geobacillus stearothermophilus*	HQ143640	99	Xm54	1/10MA+abx
XM6033	*Geobacillus stearothermophilus*	HQ143640	99	Xm56	1/10MA+abx
A42002	*Bacillus* sp. SGE47(2010)	HM566745	99	Xm49	AIA
FF8509	*Bacillus* sp. 2BSG-MG-22	AB533800	99	Xm85	FSW+FA
FF8519	*Bacillus* sp. 2BSG-MG-22	AB533800	99	Xm85	FSW+FA
FF8650	*Bacillus* sp. 2BSG-MG-22	AB533800	100	XM86	FSW+FA
FF8510	*Bacillus* sp. IBP-V002	HM021765	99	Xm85	FSW+FA
XM85109B	*Bacillus* sp. BFLP-1	FM162181	100	Xm85	FSWFA
XM4024	*Bacillus* sp. DB49(2010)	HM566930	98	Xm54	ISP2
XM4004	*Bacillus clausii*	AB251922	100	Xm54	ISP2+abx
XM3001	*Bacillus horneckiae*	HQ238937	99	Xm53	ISP2+abx
XM4002	*Bacillus licheniformis*	HM753634	99	Xm54	ISP2+abx
XM4015	*Bacillus licheniformis*	FJ493045	99	Xm54	ISP2+abx
XM4023	*Bacillus licheniformis*	HQ336641	99	Xm54	ISP2+abx
XM5010	*Bacillus licheniformis*	HQ683895	98	XmF	ISP2+abx
XM5059	*Bacillus* sp. 'Bacillus M11'	EF409318	98	XmF	ISP2+abx
XM4010	*Bacillus* sp. 1P10SA	EU977833	100	Xm54	ISP2+abx
XM1016	*Bacillus* sp. DB184(2010)	HM566896	99	Xm51	ISP2+abx
XM2001	*Bacillus* sp. DB49(2010)	HM566930	98	Xm52	ISP2+abx
XM4008	*Bacillus* sp. DB49(2010)	HM566930	98	Xm54	ISP2+abx
XM4154	*Bacillus* sp. DB49(2010)	HM566930	99	Xm54	ISP2+abx
XM4021	*Bacillus* sp. ITCr40	FR823409	100	Xm54	ISP2+abx
XM6003	*Bacillus* sp. ITCr40	FR823409	100	Xm56	ISP2+abx
XM6005	*Bacillus* sp. ITCr40	FR823409	100	Xm56	ISP2+abx
XM4022	*Bacillus* sp. MAR898	FR744809	99	Xm54	ISP2+abx
XM3005	*Bacillus* sp. RS(2010)	GU566359	99	Xm53	ISP2+abx
XM6040	*Bacillus* sp. SGE68(2010)	HM566768	99	Xm56	ISP2+abx
XM3006	*Kurthia zopfii*	DQ350826	97	Xm53	ISP2+abx
XM3006b	*Kurthia zopfii*	DQ350826	98	Xm53	ISP2+abx
XM-21-04-F	*Bacillus aryabhattai*	HQ242772	100	Xm49	MA2216
XM-21-06-F	*Bacillus aryabhattai*	HQ242772	100	Xm49	MA2216
XM-21-05-F	*Bacillus* sp. 210_11	GQ199713	99	Xm49	MA2216
XM4278	*Bacillus* sp. SV13	GU143795	99	Xm54	NTM
XM4250	*Bacillus* sp. DB49(2010)	HM566930	99	Xm54	NTM+abx
R8203A	*Bacillus safensis*	HQ284921	99	Xm82	R2AV
R8502B	*Bacillus* sp. 2BSG-MG-22	AB533800	99	Xm85	R2AV
R8306A	*Geobacillus stearothermophilus*	HQ143640	100	XM83	R2AV
R8603AF	*Geobacillus stearothermophilus*	HQ143640	100	XM86	R2AV
XM4188-F	*Bacillus flexus*	GQ279347	99	Xm54	R2AV+abx
R42104A	*Bacillus licheniformis*	HQ336648	100	Xm49	R2AV+abx
XM4189D	*Bacillus licheniformis*	JF303047	99	Xm54	R2AV+abx
XM3073D-F	*Bacillus massiliensis*	AY677116	99	Xm53	R2AV+abx
XM5112C	*Bacillus* sp. DB49(2010)	HM566930	98	XmF	R2AV+abx
XM4187A	*Bacillus* sp. RS(2010)	GU566359	99	Xm54	R2AV+abx
XM3087	*Bacillus flexus*	HM451429	99	Xm53	VL55
XM4234	*Bacillus* sp. SGE47(2010)	HM566745	100	Xm54	VL55+abx
XM4235	*Bacillus* sp. SGE47(2010)	HM566745	100	Xm54	VL55+abx
XM4226	*Bacillus* sp. SH-B27	FJ549017	99	Xm54	VL55+abx
XM6006	*Actinobacterium MH6*	HQ696527	100	Xm56	ISP2+abx
XM6002	*Brevibacillus* sp. Z0-YC6800	GQ369068	100	Xm56	ISP2+abx
XM3072E	*Actinobacterium MH6*	HQ696527	100	Xm53	R2AV+abx
XM4189C	*Brevibacillus* sp. AK-P2	HM359119	99	Xm54	R2AV+abx
XM3081B	*Brevibacillus* sp. B2(2008)	FJ268957	99	Xm53	R2AV+abx

XM3081C	*Brevibacillus* sp. B2(2008)	FJ268957	99	Xm53	R2AV+abx
XM3073A	*Bacillus massiliensis*	FN666619	99	Xm53	R2AV+abx
XM3073B	*Bacillus massiliensis*	FN666619	99	Xm53	R2AV+abx
XM4187B	*Bacillus* sp. SK-12	FN666440	97	Xm54	R2AV+abx
XM3003	*Paenibacillus lautus*	GQ284372	99	Xm53	ISP2+abx
XM4259	*Paenibacillus* sp. X6	EU236729	99	Xm54	NTM+abx
XM4184A	*Paenibacillus lautus*	HM462433	99	Xm54	R2AV+abx
XM04c78	*Staphylococcus auricularis*	NR_036897	100	XmE	ISP2+abx
XM4005	*Staphylococcus epidermidis*	FJ380964	100	Xm54	ISP2+abx
XM5013	*Staphylococcus epidermidis*	FJ605382	100	XmF	ISP2+abx
XM5060	*Staphylococcus epidermidis*	AB617573	100	XmF	ISP2+abx
XM6001	*Staphylococcus pasteuri*	HQ219847	99	Xm56	ISP2+abx
XM6012	*Staphylococcus pasteuri*	FJ217194	99	Xm56	ISP2+abx
XM4018	*Staphylococcus* sp. 6A18S5	HQ246244	99	Xm54	ISP2+abx
XM-05-01	*Staphylococcus hominis*	FJ768458	100	Xm45	MA2216
XM04c76	*Staphylococcus auricularis*	NR_036897	99	XmE	R2A
XM04c95	*Staphylococcus epidermidis*	HM218511	99	Xm49	R2A
R8501A-F	*Staphylococcus* sp. HJB003	HQ331102	100	Xm85	R2AV
R8501B	*Staphylococcus warneri*	HQ694734	99	Xm85	R2AV
XM5071A	*Staphylococcus epidermidis*	AB617572	99	XmF	R2AV+abx
XM5071B	*Staphylococcus epidermidis*	AB617573	100	XmF	R2AV+abx
XM5071C	*Staphylococcus epidermidis*	FJ613575	99	XmF	R2AV+abx
SN8101B-F	*Staphylococcus* sp. BQN2P-01d	FJ380979	99	XM81	SN
XM3083	*Staphylococcus hominis*	AJ717375	99	Xm53	VL55
XM3007	*Virgibacillus* sp. ITCr61	FR823416	99	Xm53	ISP2+abx
		Flavobacteria			
SN8106F	*Muricauda* sp. BB-My12	HM355805	95	XM81	SN
SN8104F	Mucus bacterium 28	AY654764	98	XM81	SN
SN8107F	*Coccinimonas marina* strain IMCC1846	EF108213	95	XM81	SN
		Gammaproteobacteria			
XM05c4G	*Aestuariibacter* sp. PaD1.06	GQ391977	95	Xm54	MA2216
XM05C3C1-F	*Pseudoalteromonas* sp. PC22a	HQ439550	97	Xm53	MA2216
XM4072	*Alteromonas* sp. UST061013-063	EF587997	100	Xm54	1/10MA
XM4073	*Alteromonas* sp. UST061013-064	EF587997	100	Xm54	1/10MA
XM4080	Bacterium L188*S.610*	EU935270	99	Xm54	1/10MA
XM05c6B	*Alteromonas macleodii*	HM584029	100	Xm56	MA2216
XM05c6B0	*Alteromonas* sp. UST061013-065	EF587997	100	Xm56	MA2216
XM05c6D	*Alteromonas* sp. UST061013-066	EF587997	100	Xm56	MA2216
XM05c3A	Bacterium 3D709	JF411515	98	Xm53	MA2216
XM05c6A1	Bacterium L188*S.610*	EU935270	99	Xm56	MA2216
XM3056-2	*Alteromonas* sp. UST061013-067	EF587997	100	Xm53	R2AV
XM4212	*Alteromonas* sp. UST061013-068	EF587997	100	Xm54	VL55
XM4215	*Alteromonas* sp. UST061013-069	EF587997	99	Xm54	VL55
XM4225B	*Alteromonas* sp. 46Xb1	EU440052	99	Xm54	VL55+abx
XM4225C	*Alteromonas* sp. UST061013-070	EF587997	100	Xm54	VL55+abx
XM-07-04	*Endozoicimonas* sp. R214111	FJ357696	98	XmE	MA2216
XM-20-02	*Endozoicimonas* sp. R214111	FJ357696	98	Xm49	MA2216
XM05c4E	*Endozoicimonas* sp. R214111	FJ357696	99	Xm54	MA2216
XM-07-03	*Spongiobacter nickelotolerans*	AB205011	98	XmE	MA2216
XM-07-05	*Spongiobacter nickelotolerans*	AB205011	97	XmE	MA2216
XM-20-03	*Spongiobacter nickelotolerans*	AB205011	93	Xm49	MA2216
XM05c4A	*Spongiobacter nickelotolerans*	AB205011	92	Xm54	MA2216
XM82A	*Spongiobacter nickelotolerans*	AB205011	97	XM81	MA2216
XM85102A	*Ferrimonas futtsuensis*	AB245515	98	Xm85	FSWFA
FF8116	*Gammaproteobacterium M09*	DQ399733	99	XM81	FSW+FA
FF8104	*Microbulbifer variabilis*	AB167354	99	XM81	FSW+FA
FF8104A	*Microbulbifer variabilis*	AB167354	100	XM81	FSW+FA
FF8114A	*Microbulbifer variabilis*	AB167354	100	XM81	FSW+FA
FF8665	*Microbulbifer variabilis*	AB266055	99	XM86	FSW+FA
XM2004-F	*Microbulbifer variabilis*	AB266055	100	Xm52	ISP2+abx
XM05C5E-F	*Microbulbifer variabilis*	AB167354	99	XmF	MA2216

XM85145A	Bacterium 4D713	JF411563	99	Xm85	FSWFA
XM85104B	*Photobacterium* sp. PaD2.14	GQ392002	99	Xm85	FSWFA
XM4017	Bacterium 4D713	JF411563	99	Xm54	ISP2+abx
XM4214A	*Photobacterium* sp. PaD2.14	GQ392002	99	Xm54	VL55
XM4214B	*Photobacterium* sp. PaD2.14	GQ392002	99	Xm54	VL55
XM4247A-F	*Pseudoalteromonas* sp. c42-1	EU727145	99	Xm54	1/10MA
XM4070	*Pseudoalteromonas* sp. CF14-5	FJ170035	99	Xm54	1/10MA
XM4247B	*Pseudoalteromonas* sp. OC-5	AY669166	100	Xm54	1/10MA
FF8507	*Pseudoalteromonadaceae bacterium S3*	HQ164448	100	Xm85	FSW+FA
FF8609	*Pseudoalteromonadaceae bacterium S3*	HQ164448	100	XM86	FSW+FA
FF8631B	*Pseudoalteromonas piscicida*	HM584027	99	XM86	FSW+FA
FF8516	*Pseudoalteromonas* sp. mp7	AJ551121	99	Xm85	FSW+FA
FF8603	*Pseudoalteromonas* sp. VS-4	FJ497705	100	XM86	FSW+FA
FF8615	*Pseudoalteromonas* sp. VS-4	FJ497705	99	XM86	FSW+FA
XM81117B	Bacterium 2D702	JF411484	99	XM81	FSWFA
XM81110Z	*Pseudoalteromonas prydzensis*	HM584031	99	XM81	FSWFA
XM81107X	*Pseudoalteromonas* sp. J021(2011)	JF314511	99	XM81	FSWFA
XM81107W	*Pseudoalteromonas viridis*	HQ439520	99	XM81	FSWFA
XM81107Y	*Pseudoalteromonas viridis*	HQ439520	99	XM81	FSWFA
XM05c5L	*Pseudoalteromonas piscicida*	HM584027	100	XmF	MA2216
XM05c5I	*Pseudoalteromonas luteoviolacea*	HQ439502	98	XmF	MA2216
XM05c5C1	*Pseudoalteromonas phenolica*	AB607331	99	XmF	MA2216
XM05c5J	*Pseudoalteromonas phenolica*	AB607331	99	XmF	MA2216
XM05c5D	*Pseudoalteromonas* sp. CF14-5	FJ170035	99	XmF	MA2216
XM05c5C2	*Pseudoalteromonas* sp. D5047	FJ161290	99	XmF	MA2216
XM05c5H1	*Pseudoalteromonas* sp. D6023	FJ161303	99	XmF	MA2216
XM05c5H2	*Pseudoalteromonas* sp. D6023	FJ161303	99	XmF	MA2216
SN8105	*Pseudoalteromonas* sp. VS-39	FJ497667	100	XM81	SN
R8304-F	*Pseudomonas* sp. 122	EU003535	99	XM83	R2AV
R8636-F	*Pseudomonas* sp. 122	EU003535	99	XM86	R2AV
R8647-F	*Pseudomonas* sp. 122	EU003535	99	XM86	R2AV
R8651	*Pseudomonas* sp. 122	EU003535	99	XM86	R2AV
XM3106	*Rheinheimera aquimaris*	EF076758	99	Xm53	NTM
XM05c3C	Marine bacterium Tw-3	AY028198	93	Xm53	MA2216
XM05c4H	*Shewanella* sp. MEBiC05444T	GU289647	98	Xm54	MA2216
XM4077-2	*Vibrio* sp. N104(2010)	HQ188643	99	Xm54	1/10MA
XM4241	*Vibrio* sp. PaD1.27	GQ391983	99	Xm54	1/10MA
XM4078	*Vibrio* sp. PaD3.15	GQ406648	99	Xm54	1/10MA
XM6024	*Vibrio* sp. R-14968	AJ316168	99	Xm56	1/10MA+abx
XM6027	*Vibrio* sp. R-14968	AJ316168	99	Xm56	1/10MA+abx
XM6028	*Vibrio* sp. R-14968	AJ316168	99	Xm56	1/10MA+abx
XM6031	*Vibrio* sp. R-14968	AJ316168	99	Xm56	1/10MA+abx
XM5127	*Vibrio tubiashii*	HQ890464	99	XmF	AIA
XM85114A	Bacterium 4D706	JF411556	100	Xm85	FSWFA
XM85135B	Bacterium 4D706	JF411556	99	Xm85	FSWFA
XM81104B	Bacterium 4H202	JF411587	98	XM81	FSWFA
XM81113Y	Bacterium 4H202	JF411587	99	XM81	FSWFA
XM85137	Bacterium 4H202	JF411587	99	Xm85	FSWFA
XM85138A	Bacterium 4H202	JF411587	99	Xm85	FSWFA
XM85125	*Listonella pelagia*	HM584058	99	Xm85	FSWFA
XM81113Z	*Vibrio harveyi*	HM771342	99	XM81	FSWFA
XM85103A	*Vibrio harveyi*	HM584047	99	Xm85	FSWFA
XM85117A	*Vibrio harveyi*	HM236045	99	Xm85	FSWFA
XM85117B	*Vibrio harveyi*	HM236045	100	Xm85	FSWFA
XM85120A	*Vibrio harveyi*	HM355956	100	Xm85	FSWFA
XM85105	*Vibrio owensii*	HQ908717	99	Xm85	FSWFA
XM85123	*Vibrio* sp. PaH1.05	GQ406686	99	Xm85	FSWFA
XM85123B	*Vibrio* sp. PaH1.29	GQ406714	99	Xm85	FSWFA
XM85134	*Vibrio* sp. PaH2.19b	GQ391958	99	Xm85	FSWFA
XM85126	*Vibrio* sp. PaH3.36c3	GQ406799	99	Xm85	FSWFA
XM1034	*Listonella pelagia*	HM584058	100	Xm51	ISP2+abx

XM3012	*Vibrio* sp. PaD3.12b	GQ406645	99	Xm53	ISP2+abx
XM85B	Bacterium 4D704	JF411554	99	Xm85	MA2216
XM-21-03	*Vibrio coralliilyticus* ATCC BAA-450	HM771346	98	Xm49	MA2216
XM05c5G2	*Vibrio harveyi*	HM236045	99	XmF	MA2216
XM5124	*Vibrio harveyi*	FJ161345	99	XmF	MA2216
XM83B	*Vibrio harveyi*	HM355956	100	XM81	MA2216
XM05c4C	*Vibrio* sp. S2675	FJ457461	99	Xm54	MA2216
XM4270-F	Bacterium 4D703	JF411553	99	Xm54	NTM
XM4274	Bacterium 4D801	JF411569	99	Xm54	NTM
XM4283	*Listonella pelagia*	HM584058	99	Xm54	NTM
XM3103	*Vibrio harveyi*	HM236045	99	Xm53	NTM
XM3104	*Vibrio harveyi*	HM236045	99	Xm53	NTM
XM4279	*Vibrio harveyi*	FJ161348	100	Xm54	NTM
XM4292-F	*Vibrio harveyi*	GU262992	99	Xm54	NTM
XM4271	*Vibrio mediterranei*	HM031981	100	Xm54	NTM
XM4284	*Vibrio sinaloensis* DSM 21326	HM771341	99	Xm54	NTM
XM4273	*Vibrio* sp. N104(2010)	HQ188643	100	Xm54	NTM
XM4275	*Vibrio* sp. N104(2010)	HQ188643	99	Xm54	NTM
XM4276	*Vibrio* sp. N104(2010)	HQ188643	99	Xm54	NTM
XM4281	*Vibrio* sp. S838	FJ457340	99	Xm54	NTM
XM4282	*Vibrio* sp. S838	FJ457340	99	Xm54	NTM
XM4211	*Vibrio* sp. N104(2010)	HQ188643	100	Xm54	VL55
XM4216	*Vibrio* sp. N104(2010)	HQ188643	99	Xm54	VL55
XM4224	*Vibrio harveyi*	FJ161345	99	Xm54	VL55+abx

Table S1. Cultured isolates from *Xestopsongia muta*.
