# Supplementary material for: Integration of Culture-Based and Molecular Analysis of a Complex Sponge-Associated Bacterial Community
Source: PLoS One. 2014 Mar 11;9(3):e90517. doi: 10.1371/journal.pone.0090517 (PMC3949686; doi:10.1371/journal.pone.0090517)
Supplement: Table S2 — Cultured isolates from X. testudinaria. Shown are cultured isolates from Xestospongia testudinaria, their closest relatives based on 16S rRNA gene sequence analysis (GenBank) and the isolation medium types. (PDF) [file pone.0090517.s002.pdf]

Isolate	Closest Relative	Acc. No.	% Identity	SpongeID	Isolation Medium
	Actinomycetes				
XT125-F	*Arthrobacter* sp. M1 5-4	AY762057	100	Xt01	GA+abx
XT129	*Arthrobacter crystallopoietes*	HM163531	99	Xt01	GA+abx
XT130	*Arthrobacter* sp. MH-129	AM423148	98	Xt01	GA+abx
XT223	*Arthrobacter* sp. 8A18S61	HQ246230	100	Xt02	GA+abx
XT111	*Arthrobacter* sp. 8A18S61	HQ246230	100	Xt01	R2AV+abx
XT127	*Brevibacterium* sp. SC9	EU099382	99	Xt01	GA+abx
XT132-F	*Brevibacterium iodinum*	FJ652620	99	Xt01	GA+abx
XT212	*Brevibacterium iodinum*	FJ652620	99	Xt02	GA+abx
XT218	*Brevibacterium iodinum*	FJ652620	99	Xt02	GA+abx
XT126	*Microbacterium esteraromaticum*	FJ527721	99	Xt01	GA+abx
XT128-F	*Microbacterium* sp. JL1103	DQ985063	99	Xt01	GA+abx
XT342	*Microbacterium* sp. JJD-1	FJ765512	99	Xt03	GA+abx
XT325	*Microbacterium* sp. TSWCW21	GQ284466	99	Xt03	R2AV+abx
XT327	*Microbacterium* sp. TSWCW21	GQ284466	99	Xt03	R2AV+abx
XT339	*Microbacterium* sp. TSWCW21	GQ284466	99	Xt03	R2AV+abx
XT216	*Micrococcus* sp. MG-2010-D12	FR750272	99	Xt02	GA+abx
XT226	*Micrococcus* sp. PA-E028	FJ233852	99	Xt02	GA+abx
XT320	*Micrococcus* sp. ITCr08	FR823400	99	Xt03	R2AV+abx
XT104	*Verrucosispora* sp. A1	EU714241	99	Xt01	R2AV+abx
XT110	*Micromonospora* sp. 16	FJ205721	98	Xt01	R2AV+abx
XT133-F	*Micromonospora* sp. 206203	EU437824	99	Xt01	R2AV+abx
XT207	*Micromonospora* sp. R1	EU714258	99	Xt02	R2AV+abx
XT301	*Rhodococcus equi* 103S	FN563149	99	Xt03	GA+abx
XT321	*Rhodococcus erythropolis*	DQ518913	99	Xt03	R2AV+abx
XT203-F	*Streptomyces chromofuscus*	FJ486284	99	Xt02	R2AV+abx
	Firmicutes				
XT112	*Aneurinibacillus migulanus*	AB112723	99	Xt01	R2AV+abx
XT124	*Bacillus licheniformis*	EU071553	99	Xt01	GA+abx
XT225	*Bacillus altitudinis*	JF508373	99	Xt02	GA+abx
XT230	*Bacillus subtilis*	FR846526	99	Xt02	GA+abx
XT333-F	*Bacillus licheniformis*	FJ493045	99	Xt03	GA+abx
XT316	*Bacillus* sp. 210_50	GQ199752	99	Xt03	MA2216
XT100	*Bacillus altitudinis*	JF508373	100	Xt01	R2AV+abx
XT324	*Bacillus sp. R-11590*	AJ438301	99	Xt03	R2AV+abx
XT328	*Bacillus licheniformis*	GQ375242	99	Xt03	R2AV+abx
XT336	*Bacillus licheniformis*	GQ375242	99	Xt03	R2AV+abx
XT208	*Bacterium DC36(2011)*	HQ178963	99	Xt02	R2AV+abx
XT222	*Bacterium DC36(2011)*	HQ178963	99	Xt02	R2AV+abx
XT123	*Staphylococcus pasteuri*	FJ613579	99	Xt01	GA+abx
XT306	*Staphylococcus equorum*	HQ202869	99	Xt03	GA+abx
XT341	*Staphylococcus epidermidis*	AB617572	99	Xt03	GA+abx
XT323	*Staphylococcus* sp. SB10-23	GU595329	100	Xt03	R2AV+abx
XT326	*Staphylococcus equorum*	HQ202869	99	Xt03	R2AV+abx
XT337	*Staphylococcus equorum* subsp. equorum	FR691468	99	Xt03	R2AV+abx
	Alphaproteobacteria				
XT322	*Lutibacterium anuloederans*	EU440968	99	Xt03	R2AV+abx
XT122	*Alphaproteobacterium M07*	DQ399724	99	Xt01	GA+abx
XT335	*Alphaproteobacterium JE041*	DQ097262	100	Xt03	GA+abx
XT107	Bacterium 1H203	JF411464	97	Xt01	R2AV+abx
	Gammaproteobacteria				
XT102	*Spongiobacter nickelotolerans*	AB205011	93	Xt01	MA2216
XT205	*Gammaproteobacterium MOLA 531*	AM990755	91	Xt02	MA2216
XT315	*Vibrio harveyi*	HM236045	99	Xt03	MA2216

Table S2. Cultured isolates from *Xestospongia testudinaria*.
