# Supplementary material for: Integration of Culture-Based and Molecular Analysis of a Complex Sponge-Associated Bacterial Community
Source: PLoS One. 2014 Mar 11;9(3):e90517. doi: 10.1371/journal.pone.0090517 (PMC3949686; doi:10.1371/journal.pone.0090517)
Supplement: Table S3 — OTUs shared by all individuals of X. muta and X. testudinaria, based on 16S rRNA gene sequence analysis. 70 OTUs were shared by all individuals of the two Xestospongia sp. Triangles indicate OTUs that were Xestospongia-specific. Open squares indicate OTUs that were sponge-specific. Circles indicate OTUs that were sponge/coral specific. Closed squares indicate OTUs that were not specific. Asterisks indicate OTUs that were found in a previous study of X. muta and X. testudinaria [16]. (PDF) [file pone.0090517.s003.pdf]

Classification	OTU	Number of Sequences		Classification	OTU	Number of Sequences
O JTB23 Proteobacteria*	97	204		O Nitrospina*	960	848
Δ Gammaproteobacteria	737	22		□ Nitrospina*	595	366
O Gammaproteobacteria	448	128		O Nitrospina*	72	135
□ Gammaproteobacteria	306	26		O Acidimicrobidae*	129	999
■ Gammaproteobacteria	209	28		O Acidimicrobidae	924	12
□ Gammaproteobacteria*	413	397		□ Acidimicrobidae	691	4
□ Gammaproteobacteria*	654	36		O Acidimicrobidae*	624	238
□ Alphaproteobacteria	629	84		O Acidimicrobidae	916	25
□ Alphaproteobacteria	644	26		Δ Acidimicrobidae*	838	495
□ Alphaproteobacteria*	970	173		Δ Acidimicrobidae*	835	170
O Alphaproteobacteria*	861	39		□ Acidimicrobidae*	716	53
O Alphaproteobacteria	436	71		□ Chloroflexi*	874	970
O Alphaproteobacteria*	512	230		O Chloroflexi*	932	176
O Alphaproteobacteria	408	111		□ Chloroflexi*	478	511
Δ Deltaproteobacteria*	766	47		□ Chloroflexi	496	47
□ Deltaproteobacteria*	800	96		□ Chloroflexi	425	38
O Deltaproteobacteria*	673	484		□ Chloroflexi	910	224
O Deltaproteobacteria*	678	156		□ Chloroflexi*	57	137
□ Spirochaeta*	602	75		Δ Chloroflexi*	476	41
□ Spirochaeta*	802	61		Δ Chloroflexi	339	31
□ Spirochaeta	603	60		Δ Chloroflexi	668	25
O Gemmatimondetes	619	131		Δ Chloroflexi*	152	16
Δ Gemmatimondetes*	618	281		Δ Chloroflexi	260	33
Δ Gemmatimondetes*	124	138		□ Chloroflexi	9	40
O Acidobacteria*	211	31		□ Chloroflexi*	642	88
□ Acidobacteria*	758	97		□ Chloroflexi*	486	277
■ Acidobacteria	364	109		O Chloroflexi*	416	74
□ Acidobacteria	561	28		Δ Chloroflexi*	812	46
O Acidobacteria	59	48		□ Chloroflexi	54	79
□ Acidobacteria*	883	89		O Chloroflexi*	899	282
□ Acidobacteria*	853	668		Δ Chloroflexi	394	101
Δ Candidate Division OD1	626	39		□ Chloroflexi*	881	311
Δ Candidate Division TM7*	455	12		O Chloroflexi	62	18
□ Cyanobacteria*	415	191		Δ Chloroflexi*	263	71
□ Deinococcus-Thermus*	267	24		Δ Chloroflexi*	96	17

Table S3. Classification of shared OTUs found in all four *Xestospongia* sponges.
